# Comparison between the specificity and sensitivity of the RIPASA and Alvarado Scoring systems in the diagnosis of acute appendicitis among patients with complaints of right iliac fossa

**DOI:** 10.3934/publichealth.2020001

**Published:** 2020-01-02

**Authors:** Seyed Ashkan Tabibzadeh Dezfuli, Reza Yazdani, Mohammadjavad Khorasani, Seyed Alireza Hosseinikhah

**Affiliations:** 1 Assistant Professor, Trauma and Emergency Medicine Research Center, Hormozgan University of Medical Sciences, Bandar Abbas, Iran; 2 Head of Department of Emergency Medicine, Hormozgan University of Medical Sciences, Bandar Abbas, Iran; 3 Emergency Medicine Specialist, Hormozgan University of Medical Sciences, Bandar Abbas, Iran

**Keywords:** appendicitis, RIPASA, Alvarado

## Abstract

**Introduction:**

Acute appendicitis is one of the common prevalent surgical emergencies. Various techniques, such as Alvarado Score are used for diagnosis it. This study was conducted to compare the Alvarado and RIPASA scoring systems in patients referred to Hospital with complaints of right iliac fossa pain.

**Methodology:**

This descriptive-analytic cross-sectional study was conducted in patients over 15 years with abdominal pain referred to emergency room of the Hospital. The data collection form was completed for each patient based on history and examinations and then examined by a surgeon. The pathological specimens were examined and the pathological outcomes of each patient were recorded in the relevant information collection form and finally analyzed.

**Results:**

The results for the Alvarado system showed that 42.1%, 29.2% and 28.80% of the patients had a low probability, moderate probability and high probability of appendicitis, respectively. The findings for RIPASA system showed that 19.3% of patients definitely had appendicitis. The sensitivity and specificity of the Alvarado scoring system were 53.95% and 70.18%, respectively. Positive and negative predictive values of Alvarado were 70.69% and 53.33%, respectively. In contrast, the sensitivity, specificity, and positive and negative predictive values of the RIPASA scoring system were 93.42%, 45.61%, 69.61%, and 83.87%, respectively.

**Conclusion:**

On the basis of the results, the RIPASA scoring system is a better system. Since the best cut-off point is 6 for Alvarado and 7.75 for RIPASA, it is better to use the values as a benchmark for the systems.

## 1. Introduction

Acute appendicitis is one of the most common surgical emergencies. It has been reported that incidence of appendicitis over a lifetime to be one in seven in the most people [1]. Delayed appendectomy action due to diagnostic accuracy is due to the risk of a perforated inflamed appendix and abdominal cavity infection that in turn increases the death rate [2]. Also for the definitive diagnosis of appendicitis, a pathological examination should be performed [3].

Diagnostic accuracy can be increased by using costly techniques such as ultrasound or tomography. However, these techniques may not be readily available when these are needed [4],[5]. So various techniques are used for identifying vague cases and decreasing the negative appendectomy rate, such as some scoring systems [6],[7]. The most common scoring system used in Europe and the United States of America is Alvarado Scoring system. This system is commonly used in Western countries. Very low sensitivity and specificity of the scoring system was reported in a population with a completely different ethnic origin and diet [8].

A new scoring system, so called RIPASA, is used for the population of South East Asian [9]–[11]. This system needs more parameters compared to the Alvarado system that may increase the sensitivity and specificity of the scoring system that ultimately accelerate the diagnosis of appendicitis and prevent negative appendectomy and complications of delayed appendectomy [6]. Symptoms in patients with appendicitis are similar to patients with abdominal pain especially in the early stages that make its diagnosis difficult [12]. Delayed for appendectomy due to performing accuracy experiments is associated to the risk of a perforated inflamed appendix and abdominal cavity and peritonitis infection that may ultimately cause to patient death [2]. For the definitive diagnosis of appendicitis, a pathological examination should be performed [13]. Negative appendectomy is a symptom-based surgery. Diagnostic accuracy can be improved by using techniques such as ultrasound or tomography up to about 20–40% [14]–[17], but these may not be available when these are needed [4],[5]. The different techniques are developed to identify vague cases for reducing the negative appendectomy rate [18], such as number of scoring systems designed to help early diagnosis of acute appendicitis and rapidly managing it [6],[9],[19],[20]. The most common scoring systems used in Europe and the United States of America are Alvarado Score and modified Alvarado Score [7],[20]. Scoring in these systems is performed using clinical history, physical examination, and patient tests [21]. The sensitivity and specificity of the Alvarado Score and the modified Alvarado Score have been reported to be 53–88% and 75–80% respectively [7]. However, these systems have a very low sensitivity and specificity in a population with different ethnic origin and diet [8]. Other system is RIPASA that uses more parameters such as age, gender, and duration of symptoms before referral [10].

These parameters affect the sensitivity and specificity of the Alvarado system for the diagnosis of acute appendicitis [6]. Therefore, these parameters in patients suspected of appendicitis and their effect on diagnostic accuracy has been studied in several studies [23]–[26]. This study was for first time was conducted in Iran as an Asian country and the data can be used for Iran and it's beside countries. This study was conducted aimed at comparing the Alvarado and RIPASA scoring systems in patients referred to hospital in Bandar Abbas with complaints of right iliac fossa pain.

## 2. Methodology

This prospective study was lasted for 6 months and compared the Alvarado and RIPASA scoring systems in patients referred to hospital in Bandar Abbas with complaints of right iliac fossa pain during the second half of 2017. Ethical approval for conducting the study was granted by the Hormozgan University of Medical Sciences Review Ethics Committee at Bandar Abbas, Iran (Ethics code: HUMS.REC.1396.150).

The statistical population of this descriptive-analytic cross-sectional study consisted of patients with complaints of right iliac fossa pain. Out of this population, all patients over 15 years with abdominal pain who were hospitalized in emergency room of hospital in Bandar Abbas and were willing to participate in the study were included. The data collection form was completed for each patient, based on history and examinations. Then each patient was examined by a surgeon. The appendectomy was performed on the basis of the clinical opinion of the surgeon. The pathological specimens were examined in the laboratory of hospital in Bandar Abbas. Then, the pathological outcomes per patient were recorded in the relevant information collection form.

Inclusion criteria included informed consent of the patient for participating in the study. Patients who did not want to participate in the study and did not complete the written consent were excluded. Other exclusion criteria were skin pigmentation, nail polish, venous pulse, severe anemia (hb < 5), vascular dislocation, low blood pressure, and fever that disrupt the pulse oximetry. The laparoscopic was conducted.

We analyzed the data by the Statistical Package for Social Sciences 23.0 for Windows (SPSS Inc., Chicago, IL, United States). Our findings were reported as mean and investigated within a 95% reliance and at a level of P < 0.05 significance. The Kolmogorov-Smirnov test was used for investigation of normal distribution of the quantitative data. Predicted negative appendicectomy rates for both scores were calculated and compared using Chi-square test for statistical analysis and all the variables were analyzed by unpaired student's *t*-test. In addition, receiver operating curve (ROC) at the optimal cut-off threshold scores for the RIPASA score was achieved by Stats Direct statistical software version 2.7.2 (Stats Direct Ltd, Cheshire, UK 2008).

## 3. Results

In the current study, 212 patients with complaints of abdominal pain in the right iliac fossa were examined that 133 patients (62.7%) underwent appendectomy.

The mean age was 28.3 ± 4.8 years. Of these, 56% were male. Alvarado score was calculated for each patient. Scores less than 5, between 5 and 7 and higher 8 were considered as low probability, moderate probability and high probabilities of appendicitis, respectively. Accordingly, 42.1% of the patients had a low probability, 29.2% had a moderate probability and 28.8% had a high probability of appendicitis.

Also, RIPASA score was calculated for each patient. Scores less than 5, between 5 and 7, 7.5 and 11.5 and more than 11.5 were considered as very low, low probability, high probability and definitive probabilities, respectively. Accordingly, 12.3% of patients definitely had appendicitis.

**Table 1.** Alvarado score.

		Frequency	Percent (%)
Alvarado	Low probability	89	42.1%
	Moderate probability	62	29.2%
	Highly probability	61	28.8%
	Total	212	100.0%

**Table 2.** RIPASA score.

		Frequency	Percent (%)
RIPASA	Unlikely	41	19.3%
	Low possibility	63	29.7%
	High possibility	82	38.7%
	Definitive	26	12.3%
	Total	212	100.0%

The results showed that 62.7% of patients underwent appendectomy. Accordingly, 37.3% of the patients did not undergo histologic and pathological examination for diagnosis of appendicitis. Of the patients who underwent appendectomy, 76 patients (57.1%) had positive and 42.8% had negative pathology responses.

**Table 3.** Histological results.

		Frequency	Percent (%)
Histology	Not done	79	37.3%
	Positive	76	35.8%
	Negative	57	26.9%
	Total	212	100.0%

Accordingly, the results of two scoring systems were compared in terms of definitive results from the pathological examination.

Accordingly, the sensitivity, specificity, and positive and negative predictive values were calculated for the two scoring systems.

The sensitivity of the Alvarado scoring system was 53.95% and its specificity was 70.18%. Positive and negative predictive values of Alvarado were 70.69% and 53.33%, respectively. In contrast, the sensitivity, specificity, and positive and negative predictive values of the RIPASA scoring system were 93.42%, 45.61%, 69.61%, and 83.87%, respectively.

**Table 4.** Comparison of PIPASA and Alvarado scores by pathology results.

Histology					
		Positive		negative	
		Count	Row (N%)	Count	Row (N%)
Alverado	Low probability	5	23.8%	16	76.2%
	Moderately probable	30	55.6%	24	44.4%
	Highly probable	41	70.7%	17	29.3%
Ripasa	Unlikely	1	100.0%	0	0.0%
	Low possibility	4	13.3%	26	86.7%
	High possibility	48	62.3%	29	37.7%
	Definitive	23	92.0%	2	8.0%

**Table 5.** Sensitivity, specificity, and positive and negative predictive values of RIPASA and RIPASA scoring systems.

			Alvarado (P/N)		RIPASA (P/N)	
			Positive	Negative	Positive	Negative
Histology	Positive	Frequency	41	35	71	5
		Percent (%)	70.7%	46.7%	69.6%	16.1%
	Netative	Frequency	17	40	31	26
		Percent (%)	29.3%	53.3%	30.4%	83.9%
Sensitivity (CI 95 %)			53.95% (42.13–65.45)		93.42% (85.31–97.83)	
Specificity (CI 95 %)			70.18% (56.60–81.57)		45.61% (32.36–59.34)	
Positive predictive Value (PPV) (CI 95 %)			70.69% (57.27–81.91)		69.61% (59.71–78.33)	
Negative predictive Value (NPV)			53.33% (41.45–64.95)		83.87% (66.27–94.55)	

The ROC curve was plotted for these two scoring systems.

**Figure 1.**
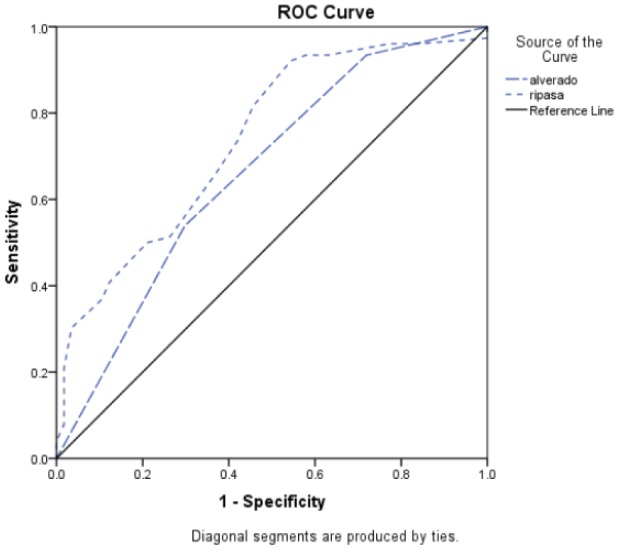
ROC curve for Alvarado and RIPASA scoring systems.

Subsequently, the area under the curve of these two scoring systems was calculated and the results were compared with each other.

The area under the curve for the Alvarado scoring system was 0.662 and it was 0.739 for the RIPASA scoring system. Given the principle that the closer number is to one, more reliable is the scoring system, the RIPASA scoring system with a significant P-value of less than 0.001 is considered to be a better technique. Also, the best cut-off point is 6 for Alvarado and 7.75 for RIPASA. The results also indicate that if the number 7.75 is considered as an interpretation benchmark for RIPASA scoring system, its sensitivity is 81.58% and the specificity is 54.39%.

These results were compared by drawing the ROC curve indicating that RIPASA with a P-value of less than 0.001 is a better technique.

## 4. Discussion

Acute appendicitis is known as one of the most usual surgical emergencies that junior doctors are encountered it. It is difficult to make a rapid and precise diagnosis for acute appendicitis. Some radiological tests such as computed tomography are broadly used and have high sensitivity (94%) and specificity (95%) for diagnosing acute appendicitis [27]. However, using a rapid and accurate diagnosis for acute appendicitis is difficult. Some practices, such as computed tomography are not economic and increase patients cost. Computed tomography not only increases costs, but may also delay emergency appendicectomy [9]. Some scoring systems such as Alvarado and the Modified Alvarado scoring are used to help a precise diagnosis of acute appendicitis in the fastest and cheapest status [20]. The systems help junior doctors for selecting emergency appendicectomy and/or conservative management [20]. The RIPASA is other scoring system that is known as a beneficial, rapid diagnostic system for acute appendicitis that only needs the patient's demographics such as age, gender and nationality, and/or a clinical history (such as anorexia, nausea and vomiting and clinical examination [9]. Scoring systems do not increase costs for patients, because the systems need only patient's demographics. The systems will not additional costs for clinical trials and these are thus economic methods for detecting appendicitis. In addition, the systems do not use apparatus with side effects; these can be accepted as safe systems.

The results of this study showed that the RIPASA scoring system is preferred. The sensitivities for RIPASA and Alvarado were 93.42% and 53.95%, respectively. The results show that RIPASA has more sensitivity. In contrast to our findings, previous studies have reported 83.01% and 81.00% for sensitivity of RIPASA and Alvarado, respectively [28]. The sensitivity for Alvarado scoring had significant difference with our findings (81% vs. 53.95%). The differences were due to studied populations. Previous studies have shown that although Alvarado scoring has good sensitivity and specificity in western population, but it has low sensitivity (50% to 59%) and specificity (23% to 94%) in Asian or oriental populations [29]–[31]. In the current study, we investigated Asian populations and our findings are in agreement with previous studies [29]–[31]. Parallel to our findings, Chong et al. [9] reported sensitivity of 97.50% for RIPASA scoring system that is closed to our findingd (93.42% vs. 97.50%). Higher sensitivity in RIPASA compared to Alvarado could be also due to more parameters in this method, because the RIPASA score uses more parameters that are not present in the Alvarado scoring, including age, gender and the duration of symptoms prior to presentation [32],[33]. The results showed that specificities of RIPASA and Alvarado systems were 70.18% and 45.61%, respectively. The results showed that if the patient is not suffering from appendicitis, Alvarado will be negative in a larger number of patients. The specificities of RIPASA in studied by Dr. Chong, Dr. Nanjundia, Dr. Karan, Dr. Boot, and Dr. Malik were 85.3%, 90.5%, 77%, 93% and 69.86% respectively, which were far higher than the calculated value in this study [23]–[26]. Sensitivity and specificity values should be higher than 80% [34],[35]. The results showed that sensitivity and specificity in Alvarado scoring was lower than 80% and it thus has not enough sensitivity and specificity. The low sensitivity of the Alvarado technique and its high level in the RIPASA technique in this study suggests that in the case of appendicitis, RIPASA is better scoring compared to Alvarado for diagnosis of acute appendicitis. This issue was significantly different in the present study than Ardam's study [28].

The results also showed that if the number 7.75 is considered as an interpretation benchmark for RIPASA scoring system, its sensitivity is 81.58% and the specificity is 54.39%. The positive predictive values of RIPASA and Alvarado systems were calculated to be 69.61% and 70.69%, respectively. These two values were very close for the two techniques. It means that if two techniques suggest the possibility of appendicitis, the final result will be positive with a relatively same probability. In contrast to our findings, the positive predictive values of RIPASA were reported to be 97.4%, 98.89%, and 94.8%, respectively in previous studies [23]. The negative predictive values for RIPASA and Alvarado systems were 83.87% and 53.33%, respectively. The results are consistent with the results of previous studies that reported the negative predictive values of RIPASA to be 95.57% and 91.8%, respectively [23],[36]. Dr Chong and their colleague [22] performed a prospective study and showed that optimal cut-off threshold score of 7.5 derived from the ROC, the sensitivity, specificity, PPV, NPV and diagnostic accuracy of the RIPASA score were 98.0%, 81.3%, 85.3%, 97.4% and 91.8%, respectively. They also showed that at the cut-off threshold score of 7.0 for the Alvarado score, the sensitivity, specificity, PPV, NPV and diagnostic accuracy were 68.3%, 87.9%, 86.3%, 71.4% and 86.5%, respectively. However, in the current study, cut-off of 6 was optimum. The differences for cut-off could be attributed to sample size. It was reported that with a sample size of > 300 patients as reported in our development phase of the RIPASA score, a predicted negative appendicectomy rate of 6.9%, a significant reduction of 9.3% was obtained [29]. A higher value in the RIPASA scoring system suggests that if the probability of appendicitis is low for the patient according to the scoring system, the patient will be less likely to have appendicitis.

## 5. Conclusion

In sum, the RIPASA scoring is commonly a much better diagnostic scoring system for acute appendicitis versus Alvarado scoring, with higher sensitivity in the Iranian population. The parameters in this scoring system can be simply obtained by completing history, and conducting a clinical examination and two simple investigations. With regards to economic dimension, the use of RIPASA scoring can decrease unnecessary inpatient admissions and expensive radiological investigations.
